# Safety and efficacy of minimally invasive posterior cervical fusion: a single center, single surgeon retrospective review

**DOI:** 10.1093/jscr/rjae559

**Published:** 2024-09-15

**Authors:** George A Crabill, Kaleb Derouen, Kierany B Shelvin, John M Wilson, Gabriel C Tender

**Affiliations:** Department of Neurosurgery, LSUHSC New Orleans, 2021 Perdido Street, 8th Floor, New Orleans, LA 70112, United States; Department of Neurosurgery, LSUHSC New Orleans, 2021 Perdido Street, 8th Floor, New Orleans, LA 70112, United States; Department of Neurosurgery, LSUHSC New Orleans, 2021 Perdido Street, 8th Floor, New Orleans, LA 70112, United States; Department of Neurosurgery, LSUHSC New Orleans, 2021 Perdido Street, 8th Floor, New Orleans, LA 70112, United States; Department of Neurosurgery, LSUHSC New Orleans, 2021 Perdido Street, 8th Floor, New Orleans, LA 70112, United States

**Keywords:** minimally invasive, posterior cervical fusion, degenerative spine disease

## Abstract

Standard posterior cervical fusion is a common surgical technique that utilizes lateral mass screws and rods for fixation. A relatively new, minimally invasive technique involving interfacet decortication and placement of spacers has shown promise in terms of outcomes. We sought to determine fusion rates and complications of this new technique at our institution to bolster current literature. We retrospectively reviewed all patients that underwent a 3-level or less minimally invasive posterior cervical fusions by a single surgeon. Patients were evaluated to determine fusion rates and postoperative complications. Twenty-eight patients underwent minimally invasive posterior cervical fusion. Twenty-seven demonstrated fusion (96%). One patient that underwent the procedure for juxta-fusional disease required additional surgery for pseudoarthrosis. The minimally invasive posterior cervical technique results in favorable fusion rates and has low complication rates. Our study strengthens current literature that this minimally invasive technique is a safe and effective alternative.

## Introduction

Healthcare costs related to spinal disorders continue to increase in the USA [1], with spinal fusions accounting for some of the most expensive operations [2]. Posterior cervical fusion (PCF) is a common surgery performed by neurosurgeons and orthopedic surgeons and is indicated for various pathologies, such as myelopathy, radiculopathy, and pseudarthrosis. The traditional, open PCF technique involves retraction of the paraspinal muscles, exposure of lamina and facets, and placement of lateral mass screws and rods. This approach results in significant soft tissue disruption, leading to increased postoperative pain and prolonging the length of hospitalization [3–5].

Minimally invasive surgical techniques have been developed to address a wide variety of spinal pathologies. These approaches promote enhanced recovery after surgery by reducing blood loss, minimizing soft tissue disruption, decreasing postoperative opioid requirements, and shortening length of hospital stay. Minimally invasive approaches to the cervical spine have lagged behind largely due to complex anatomy and lack of adoption by surgeons, however, a novel minimally invasive technique for PCF has been developed in recent years [6].

This technique involves a much smaller incision and minimizes soft-tissue injury to decorticate the facet joints and allow placement of interfacet spacers (Corus, Providence Medical Technology, Pleasanton, CA). This can be performed using fluoroscopy or navigation depending on surgeon’s preference. The interfacet spacers provide indirect foraminal decompression and stabilization while promoting fusion [7–12]. Although this technique was originally designed for patients with radiculopathy requiring foraminal decompression, we have found at our institution that this technique can provide benefit in a wider range of cervical pathology.

The aim of this study is to bolster existing literature supporting the safety and efficacy of minimally invasive posterior cervical fusion (PCF).

## Materials and methods

### Study design

This protocol was approved by the Institutional Review Board (IRB) at Louisiana State University Health Sciences Center-New Orleans (IRB #4769). This was a retrospective review of all patients that had undergone 3-level or less PCFs spine between 1 August 2012 and 30 June 2022, at our institution. The primary group included the minimally invasive interfacet fusion patients. The ancillary group for indirect comparison included the standard, open PCF patients. The primary outcome was the fusion rate, as demonstrated by either CT or dynamic X-rays at the last follow-up. The fusion was considered successful if there was clear bridging bone across the treated joint on CT and/or absence of motion at the treated joint on dynamic X-rays. We also looked for any signs of lucency around the hardware or breakage of the hardware to suggest pseudarthrosis. Complications for each group were also recorded.

### Data collection

Patients were retrospectively identified in Epic SlicerDicer between 1 August 2012 and 30 June 2022. A total of 58 patients were identified to have undergone PCF by a single neurosurgery faculty member. Demographics and clinical data were abstracted, including age, sex, medical comorbidities, and smoking status. Additionally, we collected data regarding fusion rates and perioperative parameters.

### Statistical analysis

Categorical, discrete, and continuous various were calculated with descriptive statistics. A chi-square test was performed to assess for an association between number of levels fused and pseudoarthrosis. Continuous variables were measured using a two-tailed t-test and validated with an analysis of variance (ANOVA). We utilized Excel 2023 (Microsoft), R 4.3.0, Prism 9.5.1, and SPSS Statistics version 29 (IBM) to perform statistical analysis.

### Surgical technique

**Figure 1:**
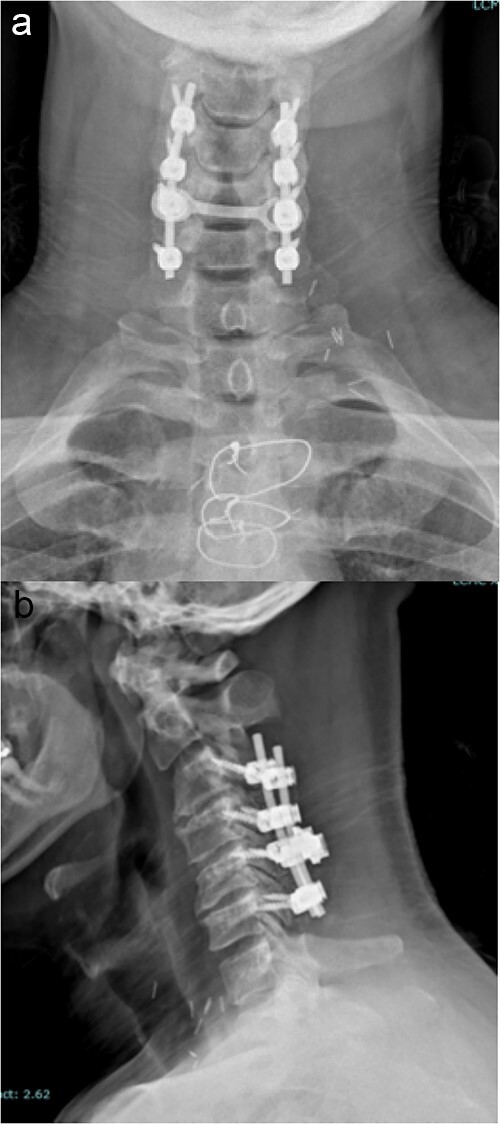
Imaging showing the instrumentation used in a standard PCF. (a) AP X-rays of the cervical spine showing the lateral mass screws and rods inserted during a standard PCF. (b) Lateral X-rays of the cervical spine showing the lateral mass screws and rods inserted during a standard PCF.

The standard, open PCF technique has been previously described. Briefly, it employs a 3–5 inch midline posterior cervical incision. The cervical fascia is opened on both sides of the spinous processes and the paraspinous muscles are detached from the lamina and facets of interest in a subperiosteal fashion. The lateral mass screws are all inserted using the following protocol: a pilot hole is made in the lateral mass 1 mm caudal and 1 mm medial from the center; a hand-drill is used to create an “up and out” trajectory, usually up to 12 or 14 mm; a 4.5 mm diameter screw is inserted in the created path. The facets are decorticated with the high-speed drill and bone graft is placed in the posterolateral gutters over the facet joints of interest. A lordotic rod is placed on top of the lateral mass screws and locked in place with set screws. If the spinal canal is stenotic, a laminectomy at the levels of interest is also performed and a cross-link is added to provide increased stability and provide a protective barrier, in case re-exploration is needed. Final images of the construct are obtained (Fig. 1).

The minimally invasive technique will be presented in further detail (Video 1).

**Figure 2:**
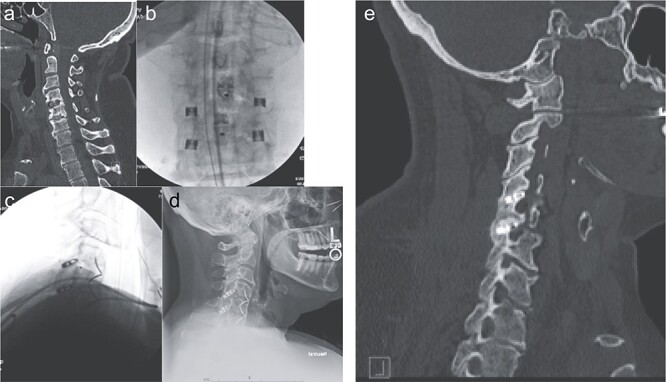
Imaging showing the minimally invasive interfacet cages technique. (a) Computer tomography imaging, sagittal view, showing lack of interbody fusion after ACDF 4-5-6. (b) AP intraoperative fluoroscopic images showing the placement of the interfacet cages. (c) Lateral intraoperative fluoroscopic images showing the placement of the interfacet cages. (d) Lateral X-ray showing a solid fusion at 1 year postoperatively. (e) Computer tomography imaging, sagittal view, showing a solid fusion at 1 year postoperatively.

The patient is placed in prone position with the face rested on a regular foam pad and a shoulder pusher in place to visualize the lower cervical segments. Two C-arms are placed in the antero-posterior (AP) and lateral positions, respectively. The posterior cervical midline skin incision is ~0.5–1 inch long and typically located around the C7 or T1 spinous process. A 10-blade is used to make two incisions in the posterior cervical fascia, slightly cranial to the skin incision, one on each side of the spinous process. Up to three levels can be treated through the same skin incision, due to the lordotic alignment of the cervical spine. The procedure is continued using the dedicated instruments, which are used a specific sequence: the access chisel, the decortication trephine, the guide tube, the decortication rasp, the fork mallet, the decortication burr, the cage inserter, and the bone graft tamp. The access chisel is used to bluntly penetrate the paraspinous muscles, targeting the facet joint of interest on both the AP and lateral fluoroscopic images. Once the tip of the access chisel reaches the posterior aspect of the facet joint, gentle tapping allows it to penetrate the posterior facet capsule and enter the joint; this is confirmed both by lateral fluoroscopy and a tactile feel. Once in the facet joint, an AP fluoroscopic image is obtained to confirm that the access chisel is in the middle of the joint, and then the chisel is advanced until its tip encounters the cranial pedicle. The decortication trephine is then inserted over the access chisel and used to decorticate the posterior aspects of the superior and inferior facets. The guide tube is inserted over the access chisel, which is then easily withdrawn. The decortication rasp and burr are then used for preparation of the interfacet space. Finally, the cage is packed with graft material of choice and inserted into the joint using the cage inserter until the tip of the cage touches the cranial pedicle. Optionally, a bone screw can be inserted through the cage and into the superior facet, to maximize stability. The cage inserter is then detached from the cage and removed. Final images of the construct are obtained (Fig. 2).

The standard grafting material was Demineralized Bone Matrix mixed with patient’s own bone marrow aspirate concentrate, harvested prior to surgery from one of the iliac crests with a Jamshidi needle. This was used in all the fusion cases, either anterior or posterior. In the posterior cervical laminectomy cases, the harvested bone was morselized and mixed with the above-mentioned graft.

## Results

Thirty-two patients underwent minimally invasive PCF with interfacet spacers between 2012 and 2022. There were 10 males and 24 females. The mean age was 53 years old, and the range was 37 to 66. Of the 32 total patients, 4 were lost to follow up and were thus excluded. All surgeries were completed by a single surgeon.

Twenty-five of the 28 patients underwent this procedure for pseudoarthrosis after a failed anterior cervical discectomy and fusion (ACDF). These patients’ symptoms transiently improved following the ACDF but later experienced severe recurrence. Imaging showed incomplete bridging bone across the ACDF levels on CT scan at 1 year postoperatively. Two patients underwent this procedure for fusion without any previous surgeries (i.e. interfacet spacers alone). One patient underwent this procedure to address adjacent segment disease.

Twenty-seven of the 28 (96%) had confirmed fusion by 1 year on follow up imaging. The fusion was considered successful if there was clear bridging bone across the treated joint on CT and/or absence of motion at the treated joint on dynamic X-rays. One patient underwent an attempted interfacet fusion for proximal segment disease at the single level (C3/4) above an existing anterior cervical fusion (ACDF C4–7). This patient unfortunately suffered from symptomatic pseudoarthrosis at the C3/4 level. A C3/4 ACDF was ultimately performed. The patient recovered without complication and demonstrated fusion at 1 year follow up.

There were no intraoperative complications, and hardware position was confirmed to be in the proper position postoperatively on cervical X-ray. One patient had a superficial skin infection postoperatively that resolved with oral antibiotics.

As an indirect comparison, there were 26 patients that underwent open PCF of three levels or less over the same time course by the same surgeon for variable pathologies. Of the 26, 24 were male and 2 were female, with a mean age was 60 years and a range of 40–73 years. Five were lost to follow up and thus excluded. Fourteen of the 21 demonstrated fusion at 1 year based on the criteria outlined above. The fusion rates for the open versus minimally invasive groups were 66 and 96%, respectively (*P* < 0.01). Seven patients exhibited lucency around at least one of the lateral mass screws, suggestive of pseudarthrosis; however, none of these patients required revision surgery during the study period.

## Discussion

Minimally invasive spine surgery is becoming more prevalent in the USA. The technique of using interfacet spacers for PCF was introduced a decade ago [6] and is showing utility in a variety of spinal pathology: cervical radiculopathy, pseudarthrosis, adjacent level disease [7, 11, 13, 14]. The interfacet cages also increase spinal stability [15, 16].

Previous studies have shown that the minimally invasive interfacet technique results in minimal soft tissue disruption and shortens length of hospitalization [12]. This is the benefit of the minimally invasive tools, which are designed to bluntly dissect the soft tissues, which minimizes postoperative pain. Moreover, the skin incision is typically <2 cm in length and the muscle dissection is carried out parallel to the muscle fibers, thus resulting in less injury.

Our study bolsters the literature that the interfacet spacer technique for cervical fusion is a safe and effective alternative to open fusion. In our study population, the vast majority underwent this procedure as a salvage technique following a failed cervical fusion. This demonstrates its utility as an alternative to promote fusion rather than a large, open anterior revision or open posterior fusion with lateral mass screws and rods.

There was one patient in our study that did not fuse, and subsequently required additional surgery. The index level was above a previous fusion construct and aimed to address adjacent segment disease. Although this has been shown to be effective in select patients, our patient ultimately pseudoarthrosed. It was the only patient that underwent this procedure for this pathology and only patient that failed to fuse. This may shed light on a patient population is not amenable to this technique but additional, more robust review of this select group is needed.

Our study has several limitations. This was a retrospective review with a relatively small sample size. Additionally, operative/hospital metrics and clinical outcomes were not evaluated in the analysis, however they have been sufficiently established. Further studies are needed to understand the long-term outcomes and complications of minimally invasive PCF technique and to identify the patient population that would benefit the most from this surgical approach.

## Conclusions

The minimally invasive PCF technique with interfacet spacers results in favorable fusion rates and has a low complication rate. This technique appears to be a useful tool in the armamentarium of spine surgeons in select cases.

## Supplementary Material

MI_Posterior_Cervical_Fusion_rjae559

Video_caption_rjae559
